# RetiNerveNet: using recursive deep learning to estimate pointwise 24-2 visual field data based on retinal structure

**DOI:** 10.1038/s41598-021-91493-9

**Published:** 2021-06-15

**Authors:** Shounak Datta, Eduardo B. Mariottoni, David Dov, Alessandro A. Jammal, Lawrence Carin, Felipe A. Medeiros

**Affiliations:** 1 Department of Electrical and Computer Engineering, Pratt School of Engineering, Duke University, Durham, NC 27708 USA; 2 Vision, Imaging and Performance (VIP) Laboratory, Duke Eye Center, Duke University, Durham, NC 27705 USA

**Keywords:** Electrical and electronic engineering, Computer science, Glaucoma

## Abstract

Glaucoma is the leading cause of irreversible blindness in the world, affecting over 70 million people. The cumbersome Standard Automated Perimetry (SAP) test is most frequently used to detect visual loss due to glaucoma. Due to the SAP test’s innate difficulty and its high test-retest variability, we propose the RetiNerveNet, a deep convolutional recursive neural network for obtaining estimates of the SAP visual field. RetiNerveNet uses information from the more objective Spectral-Domain Optical Coherence Tomography (SDOCT). RetiNerveNet attempts to trace-back the arcuate convergence of the retinal nerve fibers, starting from the Retinal Nerve Fiber Layer (RNFL) thickness around the optic disc, to estimate individual age-corrected 24-2 SAP values. Recursive passes through the proposed network sequentially yield estimates of the visual locations progressively farther from the optic disc. While all the methods used for our experiments exhibit lower performance for the advanced disease group (possibly due to the “floor effect” for the SDOCT test), the proposed network is observed to be more accurate than all the baselines for estimating the individual visual field values. We further augment the proposed network to additionally predict the SAP Mean Deviation values and also facilitate the assignment of higher weightage to the underrepresented groups in the data. We then study the resulting performance trade-offs of the RetiNerveNet on the early, moderate and severe disease groups.

## Introduction

Glaucoma is the leading cause of irreversible blindness in the world, and it is estimated that over 70 million people are affected by it^1^. Defined as a chronic and progressive optic neuropathy, it is characterized by the progressive loss of retinal ganglion cells, resulting in characteristic changes in the optic disc and consequent visual loss^2^. Although irreversible, adequate treatment can slow or even prevent further damage caused by the disease^3^. This highlights the need for early diagnosis and accurate prediction of glaucoma progression. In clinical practice, investigation and monitoring of patients with glaucoma involves evaluation of the visual field using Standard Automated Perimetry (SAP), and evaluation of the optic disc and Retinal Nerve Fiber Layer (RNFL) using Optical Coherence Tomography (OCT).

Currently, SAP is the most frequently used method for detecting visual loss due to glaucoma^4^. It is most commonly performed using the 24-2 Swedish Interactive Threshold Algorithm^5^ of the Humphrey perimeter (Carl-Zeiss Meditec, Inc.), by presenting luminous stimuli with varying intensities at 54 different locations of the visual field and recording the patient’s response to the stimuli. The patient is typically required to press a switch each time they are able to see a presented stimulus. The dimmest stimulus which is detected at least 50% of the time by the patient, in each visual field location, is recorded using a logarithmic scale, where higher values represent lower brightness, and therefore better results. Although SAP is the mainstay strategy for visual field testing, there are issues associated with the testing strategy. Due to the interactive nature of the test, there is typically a learning curve for the patient to comprehend the test instructions. Therefore, for many patients, the first few tests cannot be relied on. Further, a high level of attention is demanded of the patient during the entire duration of the test. Fatigue and lack of attention often leads to artifacts and to unreliable results. Finally, even for well-trained patients, there is high test-retest variability associated with SAP, which can preclude or delay the diagnosis or detection of disease progression^6,7^.

With the advent of a family of better ocular imaging technologies, namely the Spectral-Domain OCT (SDOCT), it has become possible to image the retina with near-histologic definition, with an axial resolution of a few micrometers. Using the SDOCT peripapillary scan, it is possible to measure the thickness of the RNFL at evenly spaced points on a circle centered around the opening of the optic disc. In contrast to SAP, SDOCT is fast, highly reproducible, and does not require any response from the patient. However, while SDOCT is capable of accurately measuring structural changes in the retina and the optic disc, it is not a direct assessment of visual function. Moreover, the SDOCT also suffers from the well known “floor effect”, where extreme structural damage beyond a certain point is no longer detectable, possibly due to the presence of residual tissues and/or due to the failure of segmentation methods^8^.

Previous works have shown that it is indeed possible to draw conclusions about the visual function based on the structural information acquired with SDOCT^9–11^. Such estimates can potentially be used as proxies for visual function, particularly for patients who are incapable of undergoing SAP. Deep learning techniques, Convolutional Neural Networks (CNNs) in particular^12^, are capable of using spatial information to identify underlying relationships that may not be easily discerned by conventional methods. Some existing studies^13,14^, have used such deep learning techniques to estimate SAP summary metrics like Mean Deviation (MD, a weighted average of the age-corrected visual field values) using information acquired with SDOCT. Other works^15–17^, more closely related to our own, have attempted to estimate pointwise sensitivities for all the locations tested by SAP, based on the SDOCT thickness values. Some studies^18,19^ have even attempted to predict the SAP sensitivities based on the raw images obtained from SDOCT. However, these previous investigations have not made use of the known topographic characteristics of the RNFL when attempting to estimate SAP data^20^.

**Figure 1:**
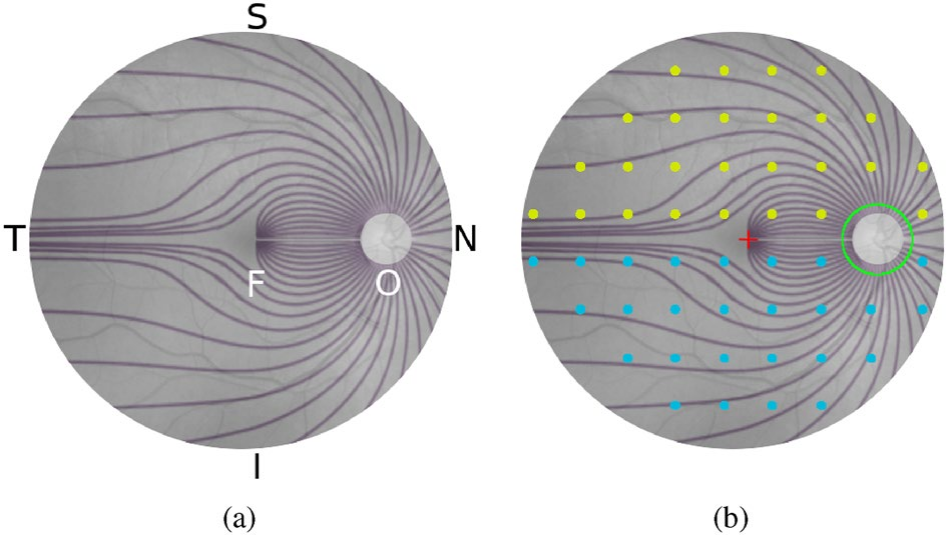
Structure of the RNFL and its relationship with the SDOCT and SAP tests (best viewed in color): (**a**) The axons from the retinal ganglion cells converge to the optic disc in a characteristic arcuate pattern while deviating from the fovea (F)^21^. The temporal, superior, nasal and inferior regions of the retina are respectively labeled T, S, N, and I while the optic disc is labeled O. (**b**) SAP measures visual function at 54 locations (2 location near the blind spot being excluded in our analysis) on the retina (cyan and yellow dots), centered (red ‘+’) at the fovea. Because of lateral inversion in the eye, the SAP locations in the superior hemiretina (yellow dots) are responsible for inferior vision while those in the inferior hemiretina (cyan dots) are responsible for superior vision. SDOCT measures the RNFL thickness at 768 equally-spaced locations along the (green) circle around the optic disc.

The RNFL is comprised of the axons of the retinal ganglion cells which are responsible for carrying visual stimuli to the optic nerve. The ganglion cells converge to the optic disc in a characteristic arcuate pattern, deviating radially from the fovea, without crossing into the opposite half of the retina (see Fig. 1), eventually emerging from the eye as the optic nerve^22^. Since our aim is to estimate visual function at different points on the retina based on the RNFL thicknesses around the optic disc, we must essentially trace-back the path along which the retinal axon fibers converge to the optic disc. We hypothesize that this approach will allow us to relate the amount of nerve tissue around the optic disc (represented by the RNFL thickness) to the appropriate SAP locations likely to be affected by it, thus improving the estimates. To this end, we propose RetiNerveNet (a.k.a. RetiNN), a deep fully convolutional neural network architecture that is inspired by this structure of retinal axon fibers. To the best of our knowledge, RetiNerveNet is the first method that incorporates elements of the structure of the retinal ganglion axon fibers for the task of estimating all the individual visual field values based on SDOCT information. Additionally, we attempt to further augment the RetiNerveNet by training it to simultaneously predict the individual age-corrected SAP values (a.k.a. Total Deviation or TD) as well as the MD values accurately and also by assigning higher weightage to the underrepresented parts of the data (since the dataset used in our experiments is dominated by SDOCT-SAP test pairs with relatively high MD values). We also study the trade-off on performance among the early, moderate and severe disease groups, resulting from tuning the weights for the two outcomes (TD and MD) as well as those for the underrepresented groups in the data.

## Results

The proposed RetiNerveNet is a deep fully convolutional neural network consisting of two similarly structured and separate sub-networks modeling the inferior and superior hemiretinae. Each sub-network, in turn, consists of four main blocks. The first convolutional block is meant to extract coarse structural information from the fine-grained RNFL thickness values. The second block is a Recursive Progression Layer (RPL, see “Methods”). Each recursive pass through the RPL is meant to model further spatial movement through the retina outwards from the optic disc. The third block extracts rich information about the outputs from the RPL by projecting onto different subspaces, while the fourth combines the different representations obtained from the former block into a single scalar output. Hence, recursive passes through RetiNerveNet sequentially yield estimates of the visual functions for locations on the retina which are progressively farther from the optic disc.

**Figure 2:**
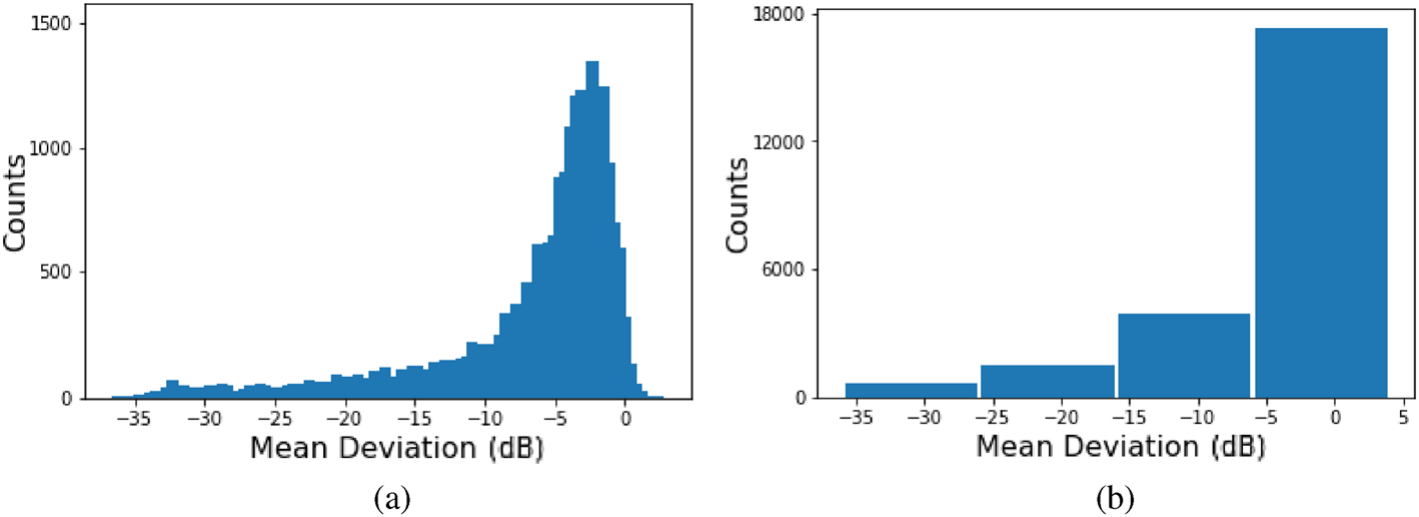
Distribution of the training data: (**a**) The data is characterized by an uneven distribution across different MD values, with a long tail towards the low MD values. (**b**) The bulk of the data has MD values better than − 6 dB. The amount of data progressively diminishes through the other intervals.

Our retrospective study is based on cross-sectional data about paired SDOCT and SAP tests, conducted within 180 days of each other. The data consisted of 38,434 pairs of tests on 23,171 eyes of 13,284 patients. 1827 of these were from 463 eyes of 235 healthy individuals, the rest being from patients having or suspected of having glaucoma. The data was split into training, validation and testing sets, respectively consisting of 23,060, 7687, and 7687 pairs of SDOCT-SAP tests, with all tests for a given patient always assigned to the same set. The training, validation as well as test data are characterized by an uneven distribution across MD values, with a long tail for the low MD values (see Fig. 2). The demographic and clinical characteristics of the data are detailed in Table 1.

**Table 1:** Demographic and clinical characteristics of the data.

Characteristic	Early/no disease	Moderate disease	Advanced disease	Overall
# SDOCT-SAP pairs	27,216	5387	5831	38,434
# SDOCT	26,860	5289	5752	37,901
# SAP	24,055	4603	4920	33,578
# Eyes	16,856	3638	3885	23,171
# Patients	10,440	3123	3062	13,284
Mean age (SD)	54.9 (20.4)	59.1 (21.2)	59.7 (20.7)	56.2 (20.7)
# Female (%)	6039 (57.8%)	1805 (57.8%)	1585 (51.8%)	9429 (56.7%)
**# Race (%)**				
Black	2831 (27.1%)	959 (30.7%)	1156 (37.8%)	4946 (29.8%)
White	6407 (61.4%)	1826 (58.5%)	1540 (50.3%)	9773 (58.8%)
Other	1202 (11.5%)	338 (10.8%)	366 (12.0%)	1888 (11.4%)
Mean RNFL thickness in μm (SD)	87.5 (17.5)	73.6 (20.9)	64.2 (23.4)	82.0 (21.0)
Median MD in dB (IQR)	− 1.6 (− 3.1, − 0.3)	− 8.2 (− 9.8, − 6.9)	− 18.8 (− 25.1, − 14.9)	− 2.7 (− 6.9, − 0.8)
Median PSD in dB (IQR)	1.9 (1.5, 2.6)	7.0 (4.6, 9.5)	9.7 (7.4, 11.9)	2.3 (1.6, 5.7)

SD: standard deviation; IQR: inter-quartile range.

The simplest baseline method for experimental comparison is a linear regression model where each of the superior (inferior) visual field values are modeled as linear functions of all the inferior (superior) RNFL thickness values, having 19,968 learnable parameters. We also compare against a fully connected neural network model in which both the superior and inferior sub-networks consist of two hidden layers with 32 nodes each and ReLU activations^23^, followed by a 26-dimensional output layer; consequently this model has 28,468 learnable parameters. To investigate the effectiveness of the recursive structure of RetiNerveNet, we also compare it against a vanilla convolutional model which employs a series of 7 successive convolutional layers to model the spatial movement outwards from the optic disc, instead of 7 recursive passes, and also does not use skip connections. This network has 27,840 learnable parameters. To put things in context, the number of learnable parameters for RetiNerveNet is 18,864.

### RetiNerveNet performance

**Figure 3:**
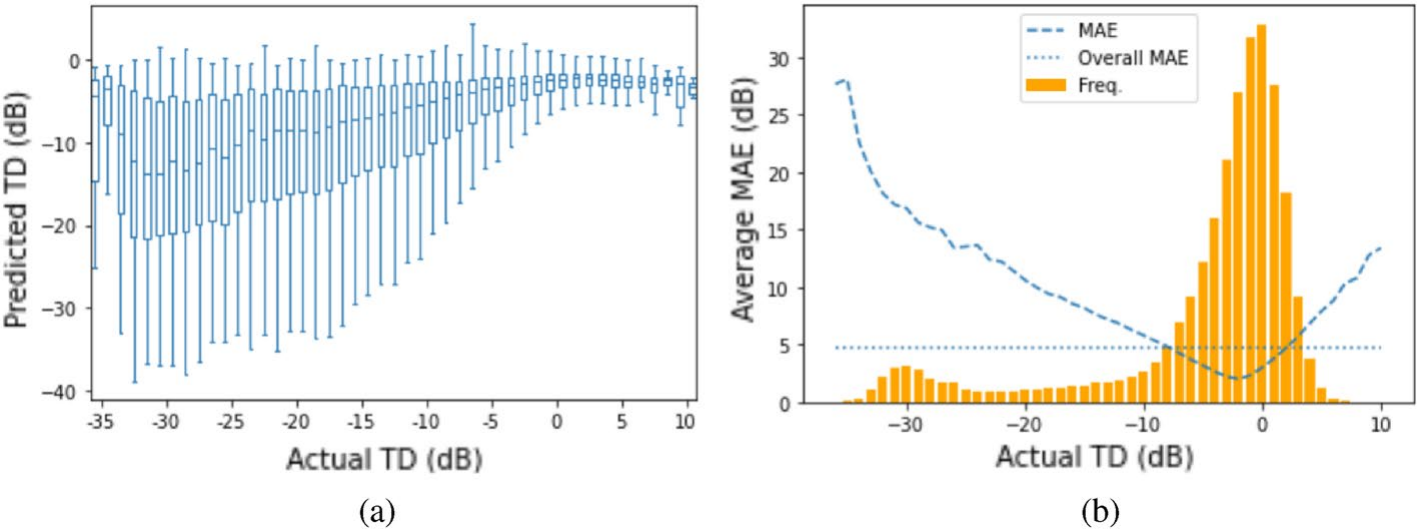
Analyses of the results obtained by RetiNerveNet (best viewed in color): (**a**) The plot of the actual versus estimated SAP TD values shows that RetiNerveNet is prone to respectively underestimating and overestimating the highly positive and highly negative TD values, with the performance being worst on the lower TD values. (**b**) RetiNerveNet is observed to be most accurate for the most frequent TD values. The error roughly in the range [− 8, 3] is observed to be lower than the overall average MAE (denoted by the horizontal dotted lines).

We compare the performance of the contending methods to reconstruct the SAP visual field values based on the SDOCT RNFL thickness values. The results are presented in Table 2 in terms of the average Mean Absolute Error (MAE) over all the 52 visual field points for the test data, with overall performance (performance on all tests) being shown alongside individual performances on tests grouped based on MD values. The tests with MD values greater than − 6 are considered to exhibit the early (or no) disease, test with MD values less than − 6 but greater than − 12 are indicative of moderate disease, while tests having MD values lower than − 12 reflect advanced disease^24^. An inspection of Table 2 shows that the linear regression model overall performs worse than the other contenders, particularly on the early/no and advanced tests. This is expected owing to the simplicity of this approach. The fully connected model and the vanilla convolutional model are able to improve upon the linear regression model on both the early and advanced tests, due to their ability to model non-linear dependencies between the RNFL thickness values and the SAP values. However, both of these methods lose some performance on the moderate tests. RetiNerveNet, on the other hand, outperforms all the aforementioned contenders on all groups. In Fig. 3, we observe that the proposed method is most accurate for the more frequent TD values and is slightly prone to underestimating the rare positive TD values while having a higher tendency to overestimate the lower TD values that are underrepresented in the data.

**Table 2:** Average MAE (with standard errors) for visual field prediction based on RNFL thickness.

Method	Early/no disease	Moderate disease	Advanced disease	Overall
Linear regression	$4.11 \; (0.01)$	$6.36 \; (0.03)$	$13.34 \; (0.04)$	$5.54 \; (0.01)$
Fully connected model	$3.38 \; (0.01)$	$6.47 \; (0.03)$	$12.65 \; (0.04)$	$5.00 \; (0.01)$
Convolutional model	$3.32 \; (0.01)$	$6.68 \; (0.03)$	$11.97 \; (0.04)$	$4.90 \; (0.01)$
RetiNerveNet	$\mathbf {3.31} \; (0.01)$	$\mathbf {6.34} \; (0.03)$	$\mathbf {11.26} \; (0.04)$	$\mathbf {4.70} \; (0.01)$

Best results in boldface.

### Performance trade-off

**Figure 4:**
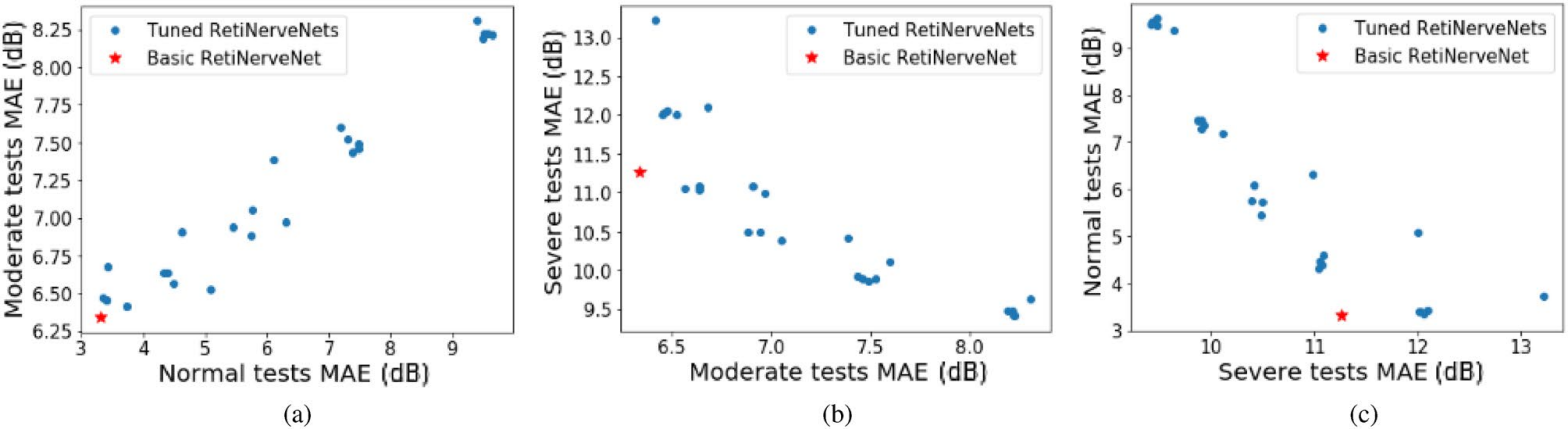
Trade-off among the performances on the early/no, moderate, and advanced disease MD groups of tests: The performance on the advanced disease group improves when we assign higher weightage to tests from the underrepresented MD intervals, while the performance on the early/no disease and moderate disease groups worsens. Very little trade-off is observed between the early/no disease and moderate disease groups. The basic RetiNerveNet performs particularly well on the early and moderate disease tests, at the expense of the advanced disease group.

As there is imbalance in the number of tests across MD values, naïvely training the RetiNerveNet results in uneven performance across the early (or no) disease, moderate disease, and advanced disease groups. Moreover, the basic RetiNerveNet also does not have a way to control how accurately the model predicts the individual TD values, as opposed to the overall MD values. Therefore, to explore the trade-offs among the groups as well as that between the TD and MD values, we make two modifications to the basic RetiNerveNet architecture. Firstly, we incorporate an additional output which simultaneously predicts the MD value alongside the age-corrected SAP outcomes. Further, we divide the data into 4 intervals based on the MD values, viz. (MD > − 6 dB), (− 6 dB ≥ MD > − 16 dB), (− 16 dB ≥ MD > − 26 dB), and (− 26 dB > MD), as shown in Fig. 2. The bulk of the data lies within the first interval, with the amount of data progressively diminishing through the latter intervals. We then also introduce a tunable loss for training the network, which can assign higher weightage to tests from the underrepresented MD groups, and can also controls the relative importance of the SAP outcome estimation and the MD estimation. We therefore obtain multiple variants of RetiNerveNet, depending on the parameters of the loss used to train the network. The effect of tuning the loss is shown in Fig. 4. The basic RetiNerveNet is observed to perform very well on the early and moderate disease tests, at the expense of the advanced disease cases. On the other hand, some variants work better on the advanced disease group, but perform worse on the early and moderate groups, and vice-versa. Consequently, it is evident from Fig. 4 that there is a clear trade-off between the performance on the advanced tests and the early and moderate tests. Sometimes, in a clinical setting, reasonable initial guesses can be made about the expected MD value based on information from previous visits. In such cases, the tuned RetiNerveNet having the lowest error around the expected MD value can be used to obtain more accurate estimates of the visual field (compared to those of the basic RetiNerveNet).

### Sectoral averages and mean deviation performance

While RetiNerveNet is trained to predict the individual age-corrected SAP values (and the MD value), we also investigate the ability of RetiNerveNet to estimate the sectoral average deviations, which are averages of the age-corrected SAP values for the different sectors of the visual field^20^. The MAEs for estimating the sectoral average deviations and the MD values for all the models are presented in Table 3. The neural models perform better than the linear regression baseline across all sectors. RetiNerveNet is observed to perform the best on all the sectors as well as for MD estimation. It is also worth noting that, for sectoral average estimation, RetiNerveNet achieves the lowest MAE on the critical central sector.

**Table 3:** Average MAE (with standard errors) for sectoral averages and mean deviation estimation.

Method	Central	Temporal	Inferior	Inferior nasal	Superior	Superior nasal	MD
Linear regression	$4.99 \; (0.01)$	$5.46 \; (0.01)$	$5.67 \; (0.01)$	$5.29 \; (0.01)$	$5.87 \; (0.01)$	$5.59 \; (0.01)$	$4.31 \; (0.01)$
Fully conn. model	$4.42 \; (0.01)$	$5.06 \; (0.01)$	$5.39 \; (0.01)$	$4.95 \; (0.01)$	$5.33 \; (0.01)$	$4.72 \; (0.01)$	$3.75 \; (0.01)$
Convolutional model	$4.41 \; (0.01)$	$5.28 \; (0.01)$	$5.06 \; (0.01)$	$4.49 \; (0.01)$	$5.47 \; (0.01)$	$4.80 \; (0.01)$	$3.63 \; (0.01)$
RetiNerveNet	$\mathbf {4.40} \; (0.01)$	$\mathbf {4.98} \; (0.01)$	$\mathbf {4.87} \; (0.01)$	$\mathbf {4.30} \; (0.01)$	$\mathbf {5.26} \; (0.01)$	$\mathbf {4.57} \; (0.01)$	$\mathbf {3.51} \; (0.01)$

Best results in boldface.

We also compare the R$^2$ values for the different sectors for RetiNerveNet with those reported recently for different deep learning methods by Christopher et al.^14^ in Table 4. While being mindful of the fact that the quoted values were found in a study that employed a dataset different from our own, it is interesting to note that the proposed method either performs much better or competitively on a majority of the sectors (viz. the central, temporal, inferior, and superior sectors), compared to more complex and rich modalities. Our method is observed to perform better across all sectors in comparison to the quoted results using the RNFL thickness modality. Furthermore, RetiNerveNet exhibits the desirable property of having comparable performance on all the sectors, including the critical central region.

**Table 4:** R$^2$ values for sectoral mean pattern deviation estimation.

Method	Central	Temporal	Inferior	Inferior nasal	Superior	Superior nasal
RNFL thickness map^a^	0.08	0.11	0.06	0.45	0.31	0.52
RNFL enface image^a^	0.09	0.12	0.26	**0.60**	**0.35**	**0.67**
CSLO image^a^	0.15	0.08	0.22	0.10	0.19	0.26
Mean RNFL thickness^a^	0.07	0.02	0.01	0.28	0.14	0.28
RNFL thickness^a^	0.07	0.01	0.02	0.36	0.17	0.31
RetiNerveNet	**0.38**	**0.24**	**0.40**	0.48	0.32	0.47

Best results in boldface.

CSLO: confocal scanning laser ophthalmoscopy.

^a^Quoted from Christopher et al.^14^.

## Discussion

We propose RetiNerveNet, a deep fully convolutional neural architecture for obtaining estimates of SAP visual field values based on RNFL thickness values obtained from the more objective SDOCT tests. Unlike existing works with similar aim^9,10,13–15,17,18^, we postulate that building our network to mimic the arcuate structure of the axons of the retinal ganglion cells can help improve performance for this task. The fact that the proposed architecture performs better than a number of baselines in Table 2 seems to corroborate our hypothesis. Moreover, RetiNerveNet also performs better than its vanilla fully convolutional counterpart, suggesting that it may be possible to model seemingly complex biological structures, like that of the retinal axon fibers, as recursive passes through a single function (the RPL in our case). It is also reassuring to observe that RetiNerveNet achieved the lower MAE on the central points of the visual field, which are known to be very important from a clinical point of view^25^. Moreover, comparison with the results reported by Christopher et al.^14^ shows that our results are characterized by more uniform performance on all the sectors of the visual field. The results from the former article seem to imply that rich modalities like an enface image or an RNFL thickness map are required to achieve good performance on this task. Our results, on the contrary, show that much of the information needed to estimate visual function may be extracted from the RFNL thickness vector, using a more sophisticated model like RetiNerveNet. However, it is important to acknowledge that the proposed techniques (as well as all baseline methods) exhibit high MAE for the advanced disease group. While the high variability of SAP thresholds for the advanced disease group^26,27^ may be a possible reason behind this, the nature of the error indicates that it is more likely to be due to the bias in measurements owing to the floor effect for the SDOCT test^8^.

Since we undertake supervised deep learning in this study, the main limitations of our work are that the predictions from our models can only be as good as visual field estimates obtained from SAP, which are known to be noisy. Moreover, as the RNFL thickness values were obtained from the SDOCT scans using the conventional SDOCT software, errors inherent to that process^28^ may also have affected our results. Additionally, the floor effect for the SDOCT test results in loss of information (structural loss beyond a certain point cannot be detected) which cannot be recovered by deep learning methods. Finally, properties of the dataset we worked with may have influenced our design choices. For example, predicting the TD values instead of the SAP thresholds was found to be more effective. However, this may be specific to our dataset as a previous study^9^ has reported much lower errors on a different dataset, while predicting the SAP sensitivity thresholds. An interesting future direction of research may be to investigate how our proposed architecture may perform in conjunction with segmentation-free RNFL thickness extraction systems based on deep learning^28^.

## Methods

Our retrospective study is based on cross-sectional data about paired SDOCT and SAP tests, from the Duke Glaucoma Registry, a database of electronic medical and research records at the Vision, Imaging, and Performance Laboratory at Duke University. Healthy individuals as well as patients having or suspected of having glaucoma were included in the study. Diagnosis of glaucoma or suspect was based on International Classification of Diseases (ICD) codes^29^. We excluded patients who underwent procedures such as panretinal photocoagulation or suffered from other diseases like retinal detachment, optic neuritis, proliferative diabetic retinopathy, etc. that could impact the RNFL thickness measurements from SDOCT, or could impact the SAP visual fields. Patients younger than 18 years were also excluded. This study was approved and informed consent was waived (due to the retrospective nature of the work) by the Duke University Institutional Review Board. All methods adhered to the tenets of the Declaration of Helsinki for research involving human participants, and the study was in accordance with the regulations of the Health Insurance Portability and Accountability Act.

### Standard automated perimetry

The SAP visual field tests were performed using the 24-2 Swedish Interactive Threshold Algorithm (SITA) protocol (Carl Zeiss Meditec, Inc., Dublin, CA^5^). We excluded unreliable tests with more than 33% fixation losses or more than 15% false-positive errors. Out of the 54 visual field values obtained using the 24-2, the two values corresponding to points around the blind spot were removed, resulting in 52 sensitivity threshold values being obtained for each SAP test. The locations in the superior hemiretina are responsible for inferior vision (and vice versa). The visual fields were also corrected for age to obtain the TD values, which were to be predicted, for the 52 locations. We choose to predict the TD values instead of directly predicting the SAP thresholds as we observed the former approach to yield slightly better results in our initial experiments.

### Spectral-domain optical coherence tomography

RNFL thickness was collected from peripapillary RNFL scans, acquired using the Spectralis SDOCT (Software version 5.4.7.0, Heidelberg Engineering, GmbH, Dossenheim, Germany). RNFL thickness values at 768 evenly spaced points on a circle having a diameter of 3.45 mm, positioned around the center of Bruch’s membrane opening (the opening of the optic disc), was obtained directly form the SDOCT software. Tests having a quality score lower than 15 were excluded, according to the manufacturer recommendations.

### RetiNerveNet

**Figure 5:**
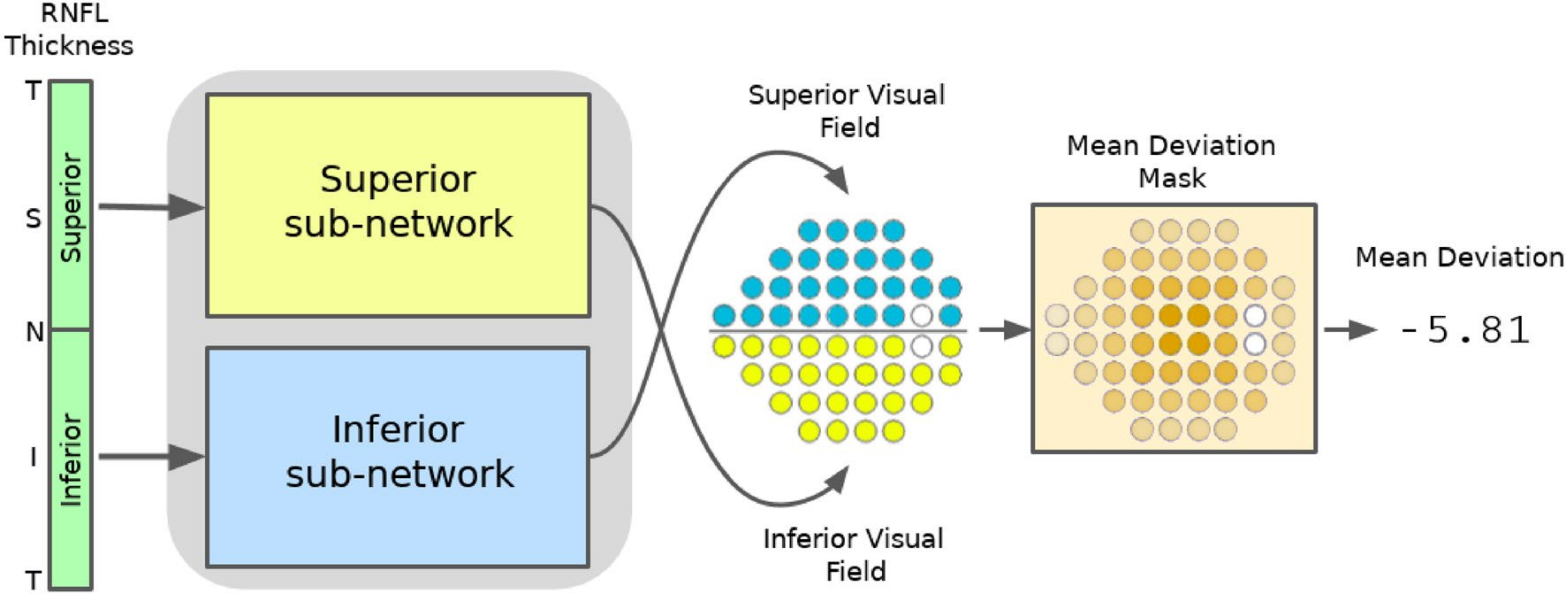
Overview of the RetiNerveNet structure: A 768-dimensional RNFL thickness vector obtained from the SDOCT test, arranged in the Temporal-Superior-Nasal-Inferior-Temporal (TSNIT) order, is split into two halves having size 384. As the retinal ganglion fibers in the eye do not cross into the opposite hemiretina, we use separate sub-networks to estimate the superior and inferior visual fields. The superior (inferior) half of the RNFL thickness values proceeds through the superior (inferior) sub-network of the RetiNerveNet to yield an estimate of the inferior (superior) half of the visual field. For the multi-task version of RetiNerveNet, a weight mask is applied to the estimated visual field to obtain a scalar estimate of Mean Deviation.

The RNFL is comprised of the axons of the retinal ganglion cells, which converge to the optic disc and emerge from the eye as the optic nerve^22^. Our task is the regression problem of estimating the 52 visual field values (excluding the 2 points around the blind spot) from the SAP test as a function of 768 RNFL thickness values obtained from the SDOCT test. To be able to do this, we must essentially trace-back the path along which the retinal axon fibers converge to the optic disc (see Fig. 1). The axons from retinal ganglion cells located in the superior half of the retina (superior hemiretina) largely do not cross to the inferior half (inferior hemiretina), and vice versa. Axons just superior to the raphe (which separates the two hemiretinae temporally to the fovea) are directed superiorly. In contrast, neighboring fibers just inferior to the raphe abruptly take the opposite direction. We assume that any minor exchange of axons between the hemiretinae nasally to the fovea can be ignored due to the coarse 24-2 grid of the SAP test. Additionally, due to the coarse resolution, we also assume that the optic disc is at zero elevation with respect to the fovea. Therefore, our proposed RetiNerveNet architecture consists of two separate, similarly structured sub-networks, viz. the superior and inferior sub-networks for respectively processing the superior and inferior halves of the RNFL thickness vector, as illustrated in Fig. 5.

Within each hemiretina, the axons converge to the optic disc in a characteristic arcuate pattern. As can be seen from Fig. 1, the axon fibers from the ganglion cells located in the temporal side of the retina do not cross the center of the fovea in their trajectory to the optic disc, but rather deviate from it radially. Additionally, it is also worth noting that the axons from the distal cells follow a path similar to the more proximal fibers but are further away from the fovea. Hence, the fibers do not cross over each other and determine a unique direction at any point on the retina. The RNFL thickness values obtained from the SDOCT test represents information at a much finer resolution than is captured by the SAP test. Further, in keeping with the fact that several axons converge into the same part of the optic disc, any defect in the optic disc is likely to impact a large number of spatially proximal ganglion cells. Consequently, each visual field point may depend on multiple RNFL thickness values in the vicinity of the point on the optic disc to which the axon in question converges. On the other hand, due to the property that the ganglion axons in the RNFL do not cross each other, ganglion cells (and therefore visual field points) that are sufficiently far apart should have low correlation. The only correlations between such distant visual field points is expected to come from factors having a global effect, such as test conditions or patient cooperation. Convolutional networks, in conjunction with pooling layers (like max pooling^30^), are effective for distilling coarse information from finer details while preserving spacial correlation^12^. Hence, we refrain from using fully connected layers (which could potentially result in spatially distant visual field points having correlated values) and instead constrain RetiNerveNet to entirely consist of 1D convolutional layers (along with max pooling). SAP measures the visual function at 52 locations on the retina, with the 26 locations in the superior hemiretina being responsible for inferior vision (and vice versa) due to lateral inversion in the eye. SDOCT, on the other hand, scans the nerve tissue along a circle around the optic disc.

**Figure 6:**
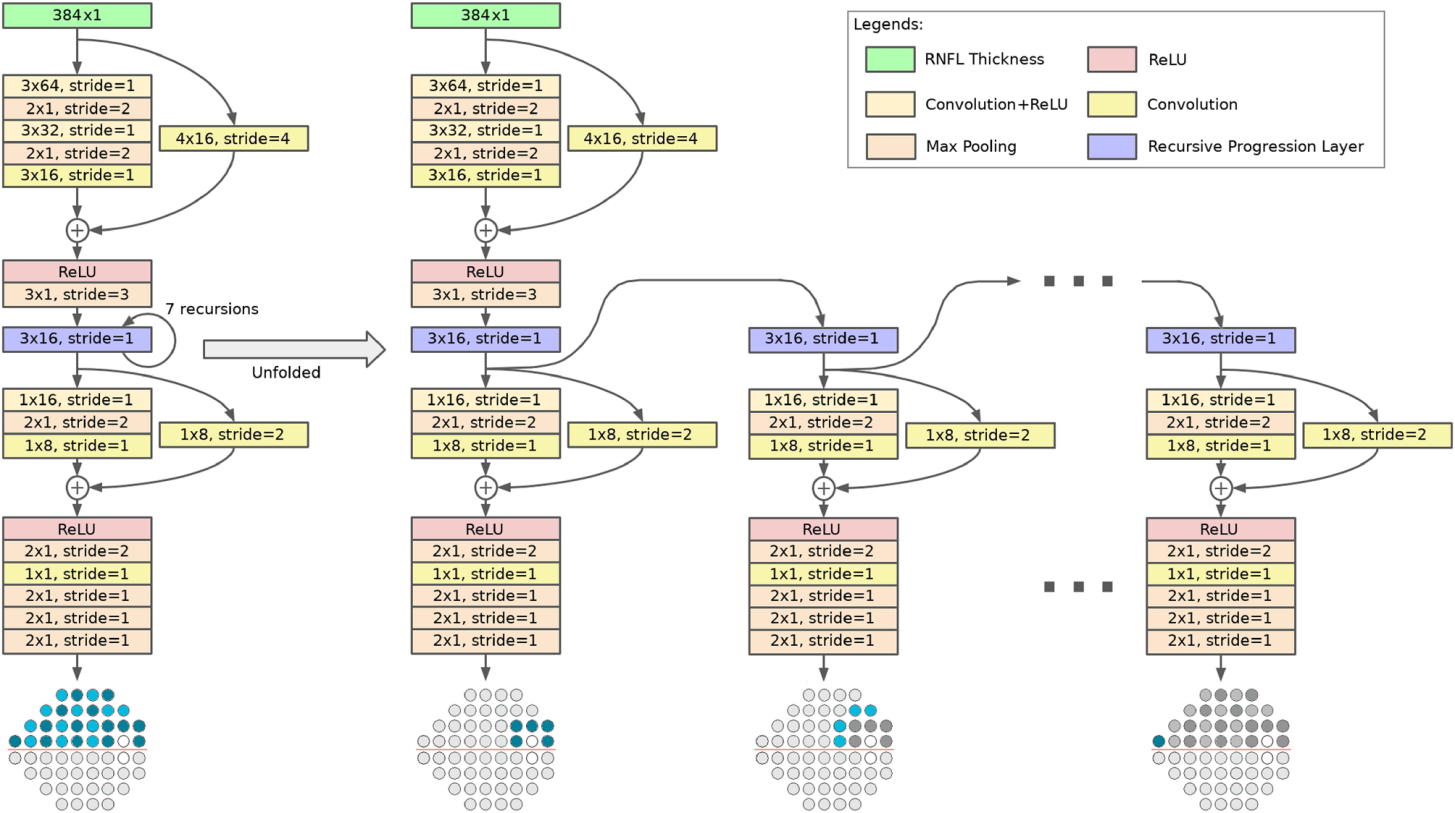
Details of the inferior sub-network of RetiNerveNet (best viewed in color): The sub-network has four main blocks. The first block consists of convolutional and max pooling layers, augmented by a convolutional skip connection. The second block only consists of the Recursive Progression Layer (RPL) which recursively models the outward spatial movement from the optic disc. None of the blocks except the RPL, correspond to any spatial movement along the retina. The next block consists of convolutional and max pooling layers, augmented by another convolutional skip connection. The final block consists of a single convolutional layer which combines the different representations obtained from the previous block into a scalar value. The subsequent max pooling layers bring down the dimension of the output to the intended size (5-dimensional vector). All convolutional layers are equipped with ReLU activations, except both the skip connections and the final convolutional layers of the corresponding blocks. The network respectively uses the first 5, 4, 4, 4, 4, 4, and 1 dimensions of the outputs after 7 successive recursive passes to obtain an estimate of the entire superior half of the visual field (consisting of 26 points). The visual field points estimated after successive passes are differentiated using contrasting shades.

The structure of the inferior sub-network is further detailed in Fig. 6. Each sub-network consists of four main blocks. The first block is composed of a series of convolutional and max pooling layers, and is meant to extract coarse structural information from the fine-grained RNFL thickness values. The second block consists of the RPL. Each recursive pass through the RPL is meant to model further spatial movement through the retina outwards from the optic disc. The third block, akin to the first, also consists of convolutional and max pooling layers. This block is meant to extract rich information about the outputs from the RPL by projecting onto different subspaces, while also further coarsening the resolution. The final block consists of a single convolutional layer followed by a series of max pooling layers. This layer combines the different representations obtained from the previous block into a single scalar value and the subsequent pooling layers reduce the resolution to the intended 5-dimensions. Deeper neural networks are known to be more difficult to train and a common solution is to introduce skip connections into the network^31^. Therefore, in order to ease the training of our proposed RetiNerveNet, we incorporate convolutional skip connections with appropriate filter size and strides in the first and third blocks of RetiNerveNet. It is important to notice that the first , third, and fourth convolutional blocks are not responsible for modeling any spatial movement. Spatial movement along the retina is meant to be modeled solely by the RPL.

#### Recursive progression layer

We trace-back the arcuate convergence of the axons fibers as multiple recursive passes through a single convolutional layer (with linear activation). In other words, the first pass through the RPL is meant to map from the SDOCT circle to the region of the retina corresponding to the first 5 visual field points nearest to the blind spot. Thereafter, farther movement away from the optic disc is modeled as subsequent passes through the same convolutional layer. To be precise, we implicitly require that the additional increment in the curvature of the axons (due to the spatial movement away from the optic disc) be captured by applying the same transformation to the output obtained after the previous pass. Thus, the second recursive pass is meant to map (from the first 5 visual field points) to the region corresponding to the next 4 visual field points closest to the optic disc, and so on (see Fig. 6). Due to the use of convolution to model this spatial movement, a small sector on the SDOCT circle can affect a larger number of visual field points as we move further away from the disc. Since RNFL axons which are wide apart in the retina are known to converge into the same region on the optic disc, this divergent property of the recursive layer is desirable for the intended trace-back. Formally, starting with ${\mathbf {r}}^{(0)} = f_{\theta _1}({\mathbf {x}})$, $f_{\theta _1}(.)$ being the first block of RetiNerveNet and ${\mathbf {x}}$ being the input RNFL thickness vector, we have

1 $$\begin{aligned} {\mathbf {r}}^{(t)} = RPL_{\theta _r}({\mathbf {r}}^{(t-1)}), \end{aligned}$$

where ${\mathbf {r}}^{(t)}$ ($t \le 7$) denotes the output after the *t*-th pass through the RPL.

While the output after the four blocks of the superior (or inferior) sub-network is always 5-dimensional, only the first 5, 4, 4, 4, 4, 4, and 1 dimensions of the output for the respective passes are deemed to be points in the visual field, as the rest are deemed to have progressed outside the region tested by SAP. Therefore, after 7 recursive passes through the RPL (and the subsequent part of the sub-network), the entire inferior (or superior) half of the visual field (consisting of 26 points) is estimated.

#### Loss function

A combination of two losses is used to train the multi-task version of the RetiNerveNet. The first loss (which alone is used to train the basic RetiNerveNet) is calculated on estimated individual visual field points, while the second loss is calculated on the estimated MD value. The loss on the estimated visual field values is

2 $$\begin{aligned} {\mathcal {L}}_{VF} = \sum _{i=1}^N \lambda _i \sum _{j=1}^{52} \rho _j (y_{i,j} - F_{\theta }^{(t_j)}({\mathbf {x}}_i))^2, \end{aligned}$$

where *N* is the number of training SDOCT-SAP pairs, $\lambda _i$ is the cost associate with the *i*-th SDOCT-SAP pair, $\rho _j$ is the cost associated with the *j*-th SAP location, $y_{i,j}$ is the true age-corrected threshold value at the *j*-th SAP location for the *i*-th SDOCT-SAP pair, $F_{\theta }^{(t)}({\mathbf {x}}_i) = (h_{\theta _3}\; \circ \;\; g_{\theta _2})({\mathbf {r}}^{(t)}_i)$ is the output of RetiNerveNet after the *t*-th recursive pass ($g_{\theta _2}$ and $h_{\theta _3}$ respectively being the third and fourth RetiNerveNet blocks), and $t_j$ is the recursive pass corresponding to the *j*-th visual field location. Further, the loss on the estimated MD values is

3 $$\begin{aligned} {\mathcal {L}}_{MD} = \sum _{i=1}^N \lambda _i (z_i - M_{\theta }({\mathbf {x}}_i))^2, \end{aligned}$$

where $z_i$ is the true MD value for the *i*-th SDOCT-SAP pair, and $M_{\theta }({\mathbf {x}}_i)$ is the corresponding estimate based on the output $F_{\theta }^{(t)}({\mathbf {x}}_i)$ from the network. Hence, the total loss used for training the RetiNerveNet is

4 $$\begin{aligned} {\mathcal {L}} = (1 - \beta ){\mathcal {L}}_{VF} + \beta {\mathcal {L}}_{MD}, \end{aligned}$$

where $\beta$ is a tunable parameter controlling the trade-off between the two losses. The cost $\lambda _i$ assigned to the *i*-th SDOCT-SAP pair on the basis of which of the 4 MD value intervals (see Fig. 2) it belongs to, is given by

5 $$\begin{aligned} \lambda _i = (1 - \alpha ) \frac{1}{N} + \alpha \frac{1}{4 N_i}, \end{aligned}$$

where $N_i$ is the number of training SDOCT-SAP pairs available from the interval to which the *i*-th pair belongs, and $\alpha$ is the hyperparameter controlling the relative importance of the underrepresented intervals. The cost $\rho _j$ associate with the *j*-th SAP location is given by

6 $$\begin{aligned} \rho _j = \frac{\exp (-d_j^2/2\gamma ^2)}{\sum _{j=1}^{52} \exp (-d_j^2/2\gamma ^2)}, \end{aligned}$$

where $d_j$ is the Euclidean distance of the *j*-th SAP location w.r.t. the center of the SAP visual field (see Fig. 1b), with unit distance between adjacent SAP locations, and $\gamma$ is a tunable parameter determining how much more important the central points of the visual field are compared to the peripheral points.

#### Experiment setup

The RetiNerveNet has three tunable parameters $\alpha$, $\beta$, and $\gamma$. Setting $\alpha =0$ and $\beta =0$ gives us the basic RetiNerveNet. We varied $\gamma$ in the set $\{0.5, 5, 50\}$ and found $\gamma =5$ to yield the lowest MAE. Therefore, we set $\gamma =5$ for all other experiments. We varied $(\alpha , \beta )$ in $G \times G$, $G=\{0.01, 0.25, 0.5, 0.75, 0.99\}$, to obtain different variants of RetiNerveNet. We conduct 5 independent runs of RetiNerveNet with each setting for a maximum of 2000 epochs. Early stopping as well as the selection of the best among the early stopped models from the independent runs is undertaken based on lowest validation loss.
